# A novel biopsy forceps assisted the removal of a deeply displaced pancreatic duct stent with pancreatic duct stenosis under the guidance of a peroral choledochoscope

**DOI:** 10.1055/a-2590-8150

**Published:** 2025-05-22

**Authors:** Jiao Tian, Yankun Hou, Tingting Yu, Lichao Zhang, Guoying Wang, Senlin Hou

**Affiliations:** 1 71213 Department of Biliopancreatic Endoscopic Surgery Department, The Second Hospital of Hebei Medical University, Shijiazhuang, China; 2 71213 Department of Intensive Care Medicine, The Second Hospital of Hebei Medical University, Shijiazhuang, China

Endoscopic removal of displaced pancreatic duct stents was challenging, especially in patients with pancreatic duct stenosis 1 . We present a novel biopsy forceps for the assisted removal of a deeply displaced pancreatic duct stent with pancreatic duct stenosis.

A 42-year-old man underwent a routine endoscopic retrograde cholangiography (ERCP) for chronic pancreatitis. Unfortunately, the pancreatic duct stent was displaced to the tail of the pancreas, and because the stent with the stenosis of the pancreatic duct could not be removed, a new pancreatic duct stent was inserted. The patient was referred to our center, where we removed the first stent with a snare ( Fig. 1 **a, b** ). Then into the peroral choledochoscope ( Fig. 2 ), a novel biopsy forceps was applied under direct vision after the pancreatic duct stent was located (SpyBite Max, Boston Scientific Corporation) grab the displaced stent and pull it to the duodenal papilla ( Fig. 3 ). It is then removed with a snare and replaced with a pancreatic duct stent ( Fig. 4 **a, b** , Fig. 5 , Video 1 ). The patient had no significant postoperative discomfort and was discharged 3 days after surgery.

**Fig. 1:**
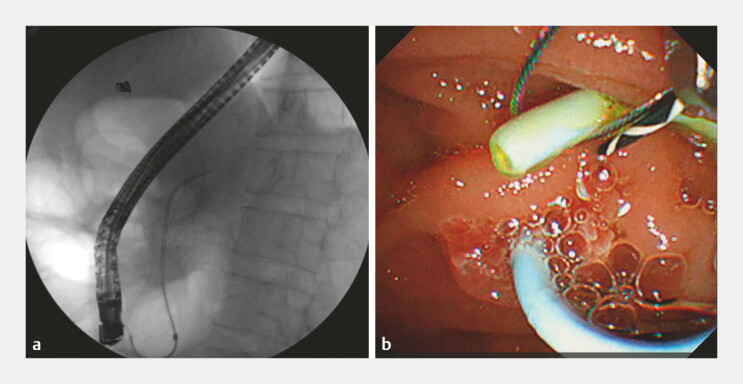
**a** The displaced pancreatic duct stent is visible below the X-ray line. **b** Remove the first bracket with a snare.

**Fig. 2:**
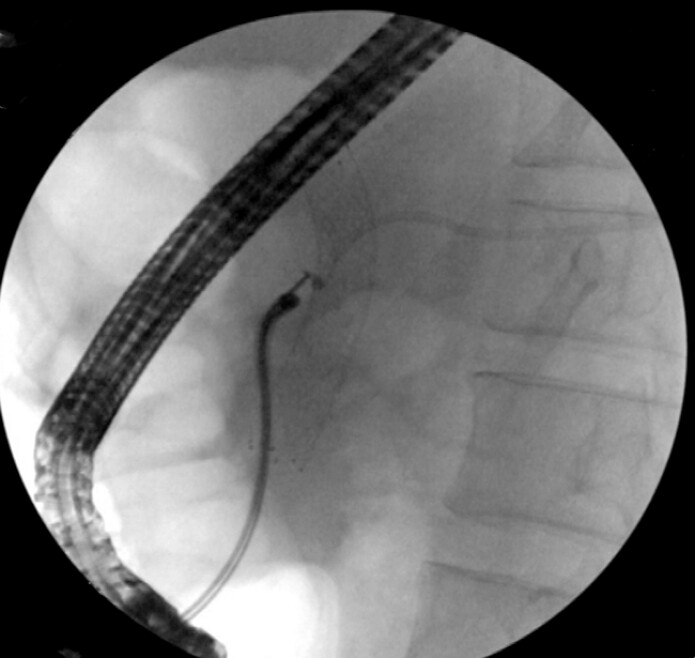
Enter the peroral choledochoscope to assist in removing the pancreatic duct stent displaced into the tail of the pancreas.

**Fig. 3:**
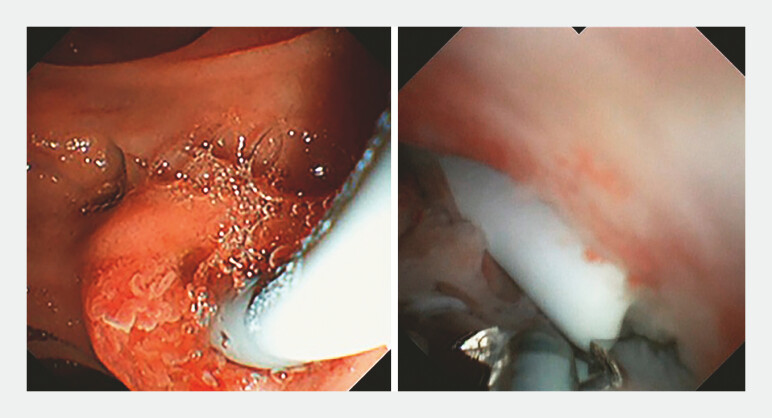
The displaced pancreatic duct stent was pulled to the duodenal papilla by biopsy forceps under the direct vision of peroral choledochoscope.

**Fig. 4:**
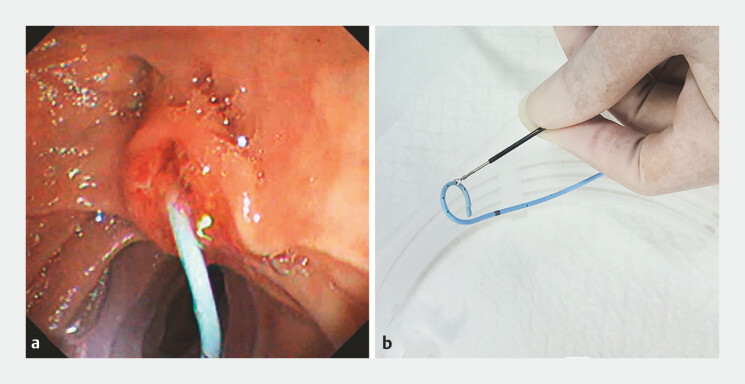
The pancreatic duct stent was pulled to the duodenal papilla and removed with a snare.

**Fig. 5:**
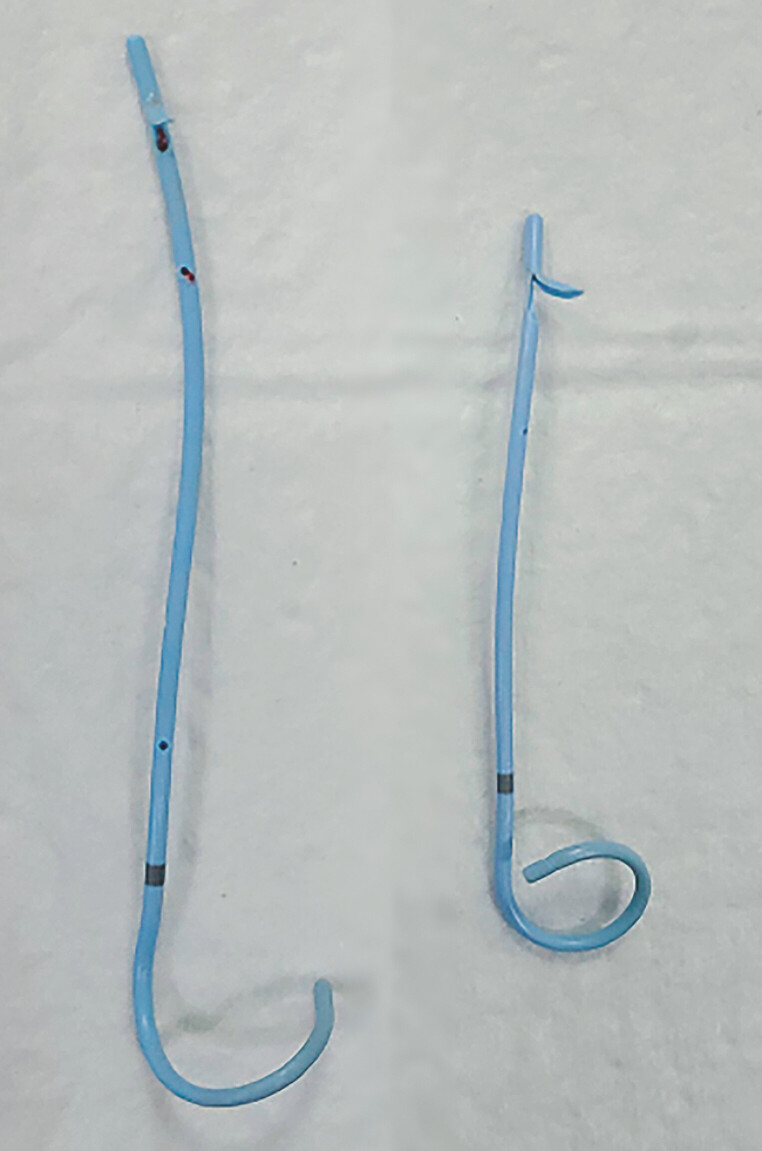
The pancreatic duct stent was removed from the body.

Novel biopsy forceps assisted the removal of a deeply displaced pancreatic duct stent with pancreatic duct stenosis under the guidance of peroral choledochoscope.Video 1

The removal of pancreatic duct stents with deep displacement has different methods in different centers 2 3 . We used a newly marketed biopsy forceps that has more rodent-shaped jaws than conventional biopsy forceps for a stronger grasp. At the same time, the biopsy forceps can be fine-adjusted repeatedly, making it easier to operate in narrow spaces. The application of the biopsy forceps provides a new way for the treatment of such patients, making it possible to remove foreign bodies in the pancreatic duct under direct vision.

Endoscopy_UCTN_Code_CPL_1AK_2AD

## Notes

Endoscopy E-Videos https://eref.thieme.de/e-videos
*E-Videos* is an open access online section of the journal *Endoscopy* , reporting on interesting cases and new techniques in gastroenterological endoscopy. All papers include a high-quality video and are published with a Creative Commons CC-BY license. Endoscopy E-Videos qualify for HINARI discounts and waivers and eligibility is automatically checked during the submission process. We grant 100% waivers to articles whose corresponding authors are based in Group A countries and 50% waivers to those who are based in Group B countries as classified by Research4Life (see: https://www.research4life.org/access/eligibility/ ). This section has its own submission website at https://mc.manuscriptcentral.com/e-videos .
